# Stiffness gradient of the beetle penis facilitates propulsion in the spiraled female spermathecal duct

**DOI:** 10.1038/srep27608

**Published:** 2016-06-23

**Authors:** Alexander E. Filippov, Yoko Matsumura, Alexander E. Kovalev, Stanislav N. Gorb

**Affiliations:** 1 Department Functional Morphology and Biomechanics, Zoological Institute of the Kiel University, Am Botanischen Garten 9, D-24118 Kiel, Germany; 2 Donetsk Institute for Physics and Engineering, National Academy of Science, 340114, Donetsk, Ukraine; 3 Department of Biology, Keio University, 4-1-1 Hiyoshi, Yokohama 223-8521, Japan

## Abstract

It is well known that sexual selection is the main driving force of substantial diversity of genitalia found in animals. However, how it facilitates the diversity is still largely unknown, because genital morpho/physical features and motions/functional morphology of the structures in sexual intercourse are not linked for the vast majority of organisms. Here we showed the presence of material gradient and numerically studied an effect of stiffness gradient of the beetle penis during its propulsion through the female duct. We found that stiffness gradient on the penis essentially affects its propulsion. Microscopic investigation suggests the possibility that the tip of the hyper-elongated penis is softer than the rest of it, and our numerical model confirms that this type of distribution of stiffness gradient aids in faster propulsion than other types. This result indicates that previously ignored physical properties of genital materials are of crucial importance in evolutionary studies of genitalia.

Male and female genitalia evolve more quickly and yield remarkable diversity in comparison with other characters^1^,^2^,^3^,^4^,^5^. This phenomenon has previously attracted attention of evolutionary biologists, and its mechanisms have been intensively investigated by sophisticated experiments^4^,^6^,^7^,^8^,^9^,^10^,^11^,^12^,^13^,^14^,^15^. It is widely accepted that the immense diversity of genital structures is driven by sexual selection in general^1^,^2^,^5^,^16^. Moreover most researches in this field were focused on the male genitalia^17^, however, it has been recently uncovered that females play an active role in sexual selection by active choice of males^18^,^19^ and that interactions between sexes are very important factors for genital evolution of both sexes^20^,^21^.

Knowledge of how male and female genital properties affect physical interaction during sexual intercourse is essential in the evolutionary studies of genital structures. Since natural selection does not act directly on shapes, but rather influences shapes indirectly through functions, links between genital morpho-physical features and their functional significance are critically important to understand evolutionary mechanisms of diversification. Nevertheless biomechanics which challenges comprehensive understanding of kinematics and dynamics of movements in relation to the structural features have been largely ignored in this field, but recently this topic started to contribute more to our knowledge due to modern experimental techniques, elaborate modeling tools, and by increasing number of interdisciplinary studies^15^,^22^,^23^,^24^,^25^,^26^,^27^.

Hereby we numerically modeled hyper-elongated male and female genitalia especially focusing on physical properties observed in cassidine beetles, to gain insights about how genital features affect penile propulsion that is an initial and vital process of male and female physical interactions during copulation. In these beetles, the male penises represent an elongated cuticular flagellum, transferring sperm, and female genitalia where the flagellum is inserted represent a partly cuticular and helical spermathecal duct including some reversal turns^28^ called knots (Fig. 1). Recently impacts of the shapes of the female spermathecal duct on male penile propulsion were numerically evaluated in this system^24^. Moreover for insect cuticular and fibrous structures, tenent setae of beetle tarsi were demonstrated to have longitudinal material and stiffness gradients^29^, which result in specific mechanical behavior of the entire attachment system^30^. Therefore, stiffness properties of the male flagellum and female spermathecal duct were hypothesized to be an important parameter, which might influence flagellum motion within the female spermathecal duct.

In order to test this hypothesis, we analyzed the autofluorescence composition of the genitalia based on the differences of different biopolymers using confocal laser scanning microscopy^31^. This method provides indications for the material composition of arthropod cuticle structures^31^. The effect of the estimated properties on the genitalia propulsion was then evaluated using the numerical model previously established by Filippov *et al*.^24^ with certain modifications. The question of this study is how the male-female interactions during copulation are affected by the material properties of the genitalia. In the present paper we report on the stiffness gradient of the male flagellum along the duct, whereas no gradient was observed in the internal wall of the female spermathecal duct. This type of material distributions in the male and female genitalia showed the highest velocity in the numerical simulation, if compared to other variants of distributions of material properties.

## Results

### Autofluorescence composition along the flagellum

**Figure 2:**
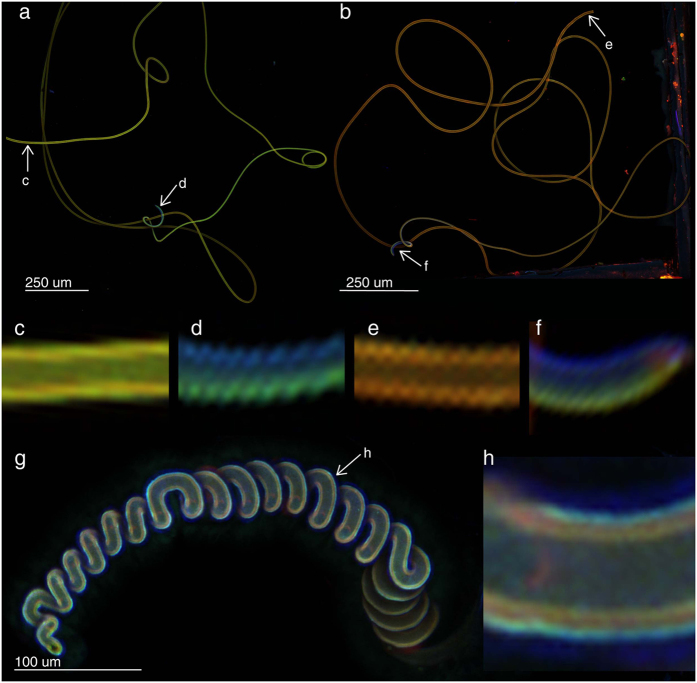
Confocal laser scanning micrographs of flagella (for two individuals **a,b**, respectively) and spermathecal duct (**g**) of *Cassida rubiginosa*. (**c–f**) The details of the base (**c,e**) and the tips (**d,f**) of the flagella are digitally enlarged from the original images (**a,b**), respectively. The stripes are artifacts of image processing. (**g**) The spermathecal duct, the left end corresponds to the beginning of the spermatheca and the right end corresponds to the beginning of the vagina. (**h**) The details of the duct. We visualized the emitted wavelengths 420–480, ≥490, ≥560, and ≥640 with blue, green, red and red, respectively.

The confocal laser scanning microscopy analysis revealed a gradient in the autofluorescence composition along the flagellum (Fig. 2). Except for the apical regions, the flagellum exhibited strong green and red autofluorescences. The tips (nearly 1% of the total length: ca. 82 μm, 97 μm, 127 μm) of the flagellum showed mainly blue autofluorescence inside the curve and green autofluorescence on another side (Fig. 2d,f). In the material of the spermathecal ducts of the females no longitudinal gradient in the autofluorescence composition was found (Fig. 2g), but we revealed a transversal gradient in the autofluorescence composition (Fig. 2h). The internal wall showed mainly orange to green autofluorescence and the external layer shows the dominance of the blue autofluorescence. The duct showed blue autofluorescence in the outer part of the duct wall and mainly green and partially red autofluorescence in the inner part of the wall (Fig. 2g,h). Same tendency was observed, when we analyzed male and female structures simultaneously, but the females showed stronger intensity of green and red autofluorescence in comparison to that of the male (Supplementary Fig. 1).

### Numerical simulation

**Figure 3:**
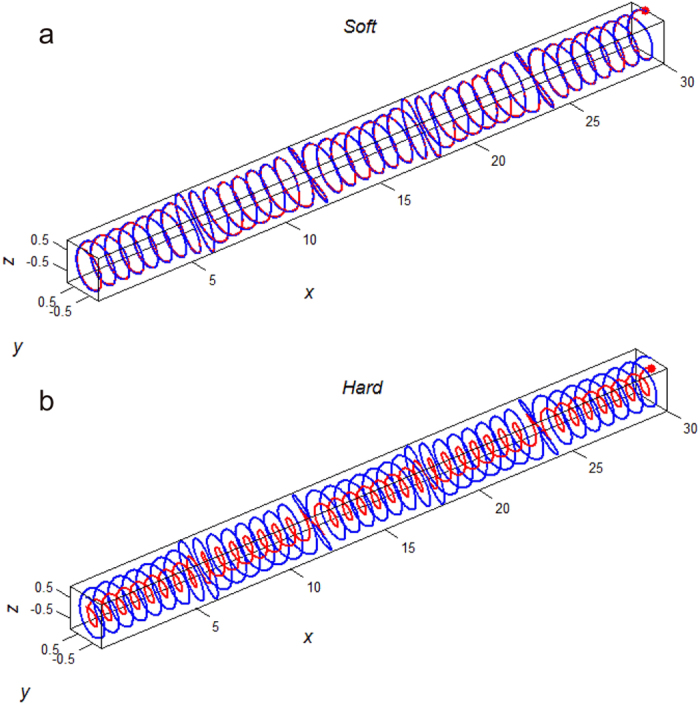
3D scheme of numerical experiments. Two extreme cases of the male flagellum stiffness are presented. (**a**) continuously soft. (**b**) continuously hard. The central axis of the female duct is shown by the blue line, the male flagellum is shown by the red line. The entire flagellum is inserted in the female spermathecal duct.

**Figure 4:**
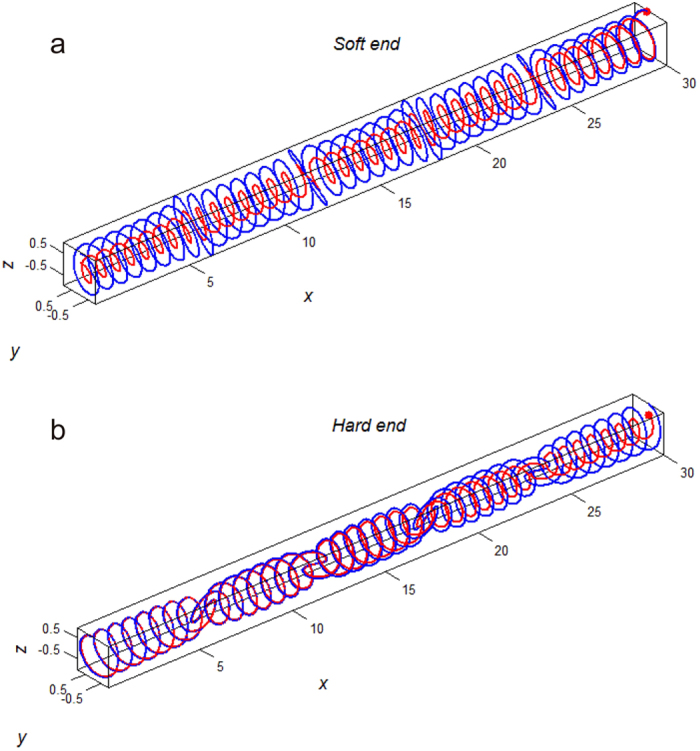
3D scheme of numerical experiments. Other two cases of the male flagellum stiffness are presented as in the Fig. 3. (**a**) the flagellum gradually softening from its basis (left side) to its end (“Soft end”). (**b**) the flagellum gradually hardening from its basis to its end. The central axis of the female duct is shown by the blue line, the male flagellum is shown by the red line. The entire flagellum is inserted in the female spermathecal duct.

We hereby applied the previous numerical model^24^ in which we numerically created the fibrous male flagellum as an elastic uncompressible fiber and the highly helical spermathecal duct as an elastic channel (Figs 3 and 4; females: blue lines, males: red lines). Although the female spermathecal duct is not only helical but also the whole helical duct is convoluted in reality, not straightened^24^, we created the female model excluding the convoluted shape of the whole helical duct as shown in Figs 3 and 4. For females, we included the knots on the duct, which were demonstrated to increase the male energy expenditure for penile propulsion in the previous study^24^ (Figs 3 and 4). The end of the male flagellum was just pushed to propel it into the female spermathecal duct to imitate the genital motion during penile penetration.

**Figure 5:**
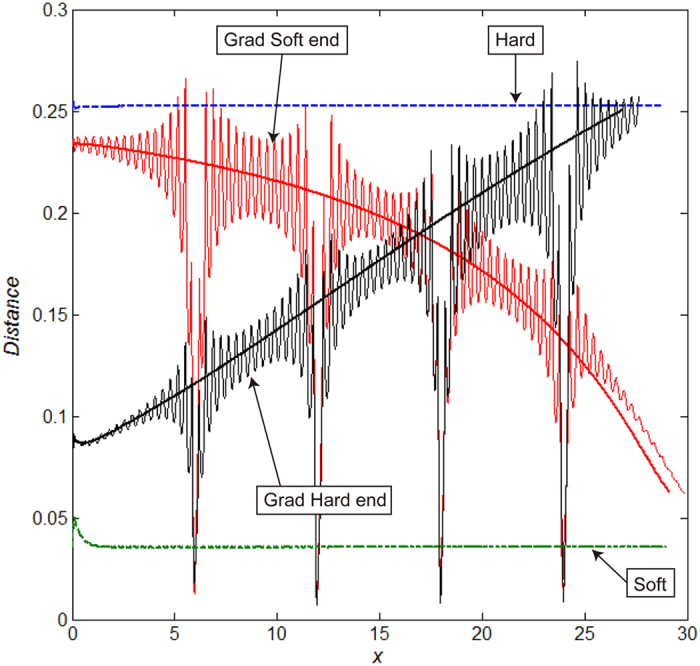
Local deviation distances of a male flagellum from a female duct axis along the x-coordinate of the channel for four cases presented in Figs 3 and 4. Corresponding cases are marked by arrows. Smooth curves represent cases for the channels without knots. Oscillating curves show the distances for the cases when male flagella had gradient and female channels had four knots.

**Figure 6:**
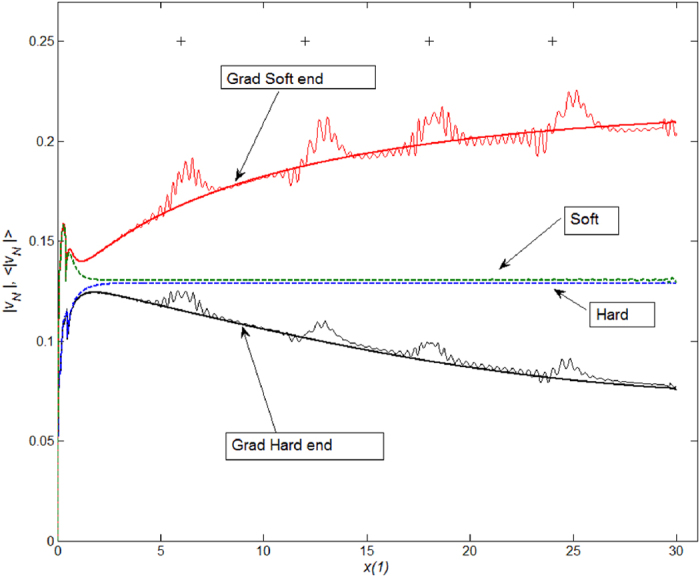
Correlation between maxima in the velocity of male flagellum tip along the female channel coordinate *x*(1) and the locations of the knots (shown with cross symbols “+”). The curves corresponding to male flagella with stiffness gradient and female channels with four knots are oscillating lines. The curves corresponding to flagella with stiffness gradient and channels without knots are shown as thick lines. Finally, the curves corresponding to flagella without stiffness gradient and female channels without knots are shown as dashed lines. The tip velocity curves are averaged over moving time window with the width corresponding to the time interval between neighboring minima.

Results of the simulations for the flagellum with uniform and gradient of rigidity (Figs 3 and 4) are summarized in Figs 5 and 6. Because the longitudinal material gradient was found only in the male flagellum and the inner wall of the female spermathecal duct showed constantly green to red autofluorescence, we focused on the analysis of the material gradient of the male flagellum below. First of all we analyzed the local deviation of the flagellum from the intact female duct axis along the x-coordinate of the duct for 4 cases: constantly soft, constantly hard, almost constantly soft with the hard end, and almost constantly hard with the soft end (Fig. 5). Each curve in Fig. 5 corresponds to the flagella rigidity distribution depicted in the Figs 3 and 4. There is a clear difference between the curves obtained for the female systems without and with the knots. This difference between them is mainly due to the various degrees of oscillations caused by the phase shift of the knots between segments of the flagellum and female duct having the same index *j*, which is produced by the phase shift around every knot *j*.

A correlation between maxima of the absolute tip velocity along the female duct and positions of the knots is presented in Fig. 6. Using Eq. 2 in the materials and methods, instant velocity of the flagellums’ tip was calculated for each case as a function of time. However, for the comparison, this velocity is plotted here as a function of the tip coordinate x(1). Every curve here presents the velocity averaged over moving time window with the width comparable to the time interval between neighboring minima. The averaging makes the maxima in velocity more pronounced. Thick lines in the figure show the velocities calculated for the same systems without knots. The propulsion velocity strongly depends on the stiffness of the flagellum. The velocity for the rigid flagellum with soft end is much higher than the same value for all other cases. Accelerated flagellum propulsion was observed at the knot sites.

## Discussion

The CLSM results of the present study indicate longitudinal gradient in the material composition of the flagellum and the absence of such a longitudinal gradient in the female spermathecal duct. According to Michels and Gorb^31^, orange (which is a mixture of the green and red) to green autofluorescence and no autofluorescence gradient along the female spermathecal duct, observed in the inner wall, suggest that the inner wall is almost homogeneously composed of sclerotized cuticle. On the contrary, the observed autofluorescence compositions of the flagellum suggest that larger parts of the flagellum are mainly composed of sclerotized cuticle, while the flagellum tip contains mainly non-sclerotized cuticle and relatively high proportions of the rubber-like protein resilin, whose concentration increases towards distal end. For other cuticular outgrowths, such as hairy attachment pads reported for many insects^32^,^33^, correlations between material and stiffness gradients were demonstrated recently^25^,^29^. A simulation showed that such a stiffness gradient, found in the hairy attachment system, affects the mechanical behavior of the hairs during contact formation/breakage^24^. In addition to it, the female structures, which we have analyzed simultaneously in the same CLSM scan with the male structures, had generally stronger intensity of green to red autofluorescence, which suggests larger thickness of the cuticular wall in females. These results inspired us to hypothesize that the beetle flagellum also may have a stiffness gradient and the inner wall of the spermathecal duct is constantly stiffer than the flagellum. Our model implemented those male and female genital properties clearly demonstrated that the stiffness gradient along the flagellum impacts flagellum’s penetration in the relatively stiff helical-like counterpart of the female.

The flagellum that is equally stiff along its length results in a greater deformation between the original and penetrated positions of the female duct (Fig. 5). It means the flagellum simply “ignores” the duct’s shape and its resultant mechanical disturbance which was demonstrated in Filippov *et al*.^24^. Nevertheless, the absolute velocity of the flagellum is the slowest in this condition (Fig. 6). Contrary to these results, the hard flagellum with the soft tip, possibly reflecting the real beetle system, shows quite different results (Figs 5 and 6), especially the velocity. It seems that the soft end of the flagellum is flexible enough to quickly adjust small curvature in the duct, and at the same time, it is strongly pushed by the rigid basal part. In reality, not only the longitudinal stiffness gradient, but also a transverse stiffness gradient observed in the apical region of the flagellum, i.e. the relatively soft inner curve and the rigid outer curve, would aid the flagellum tip to adjust to the small curvature of the female duct. Interestingly, the uniform soft flagellum and almost uniform soft flagellum with the stiff tip show worse performances in comparison with the rigid flagellum with the soft end, i.e. very big deformation of the female duct and/or much slower velocity of the male flagellum (Figs 5 and 6). It is conceivable that a flagellum with lower transversal stiffness strongly absorbs propulsion forces that in turn decrease the degree of further propulsion. Thus, the results obtained from our simulations, strongly support the hypothesis that the stiffness gradient in the beetle flagellum facilitates penile propulsion.

In our previous study^24^, the effects of variable shapes of female ducts on the penile propulsion were analyzed. It was found that certain parameters of the female duct, such as the presence of knots and the small curvature of the duct, affect the velocity decrease of the penile propulsion or the increase of the energy expenditure for propulsion. Because sexual intercourses in the field can be disrupted by other males or by natural enemies, it is highly likely that males inserting a penis and ejaculating quickly can be preferred in the course of sexual selection. Therefore, the stiffness gradient of the flagellum capable of quick penetration of the female duct can be selected as a favorable structure for both males and females, even if it could increase the energy expenditure for male penile propulsion which was demonstrated in the previous study^24^.

There are several sophisticated experimental studies investigating driving and stabilizing mechanisms of hyper-elongation^7^,^13^,^15^,^28^,^34^,^35^. However, neither of the authors took into account the material properties of genitalia. It has been recently shown that the males with the cut off tip of the flagellum in bugs acquired lower paternity^15^. This result might be partly explained by the results of our simulation. It is likely that the absence of the softer tip causes troubles for sexual behavior, because of a less effective propulsion, although the question why the slower insertion causes lower paternity in the lab condition must be considered in further details. As Dougherty *et al*.^15^ pointed out, the functional morphology has been largely ignored in the studies of genital evolution. The hyper-elongated genitalia are ubiquitous in animals, not only in insects but also in vertebrates like ducks^36^, and it is very interesting to broaden studying taxa for comparing the mechanical properties among animals. Our approach shed light on the importance of studying materials’ properties with the combination of experimental and numerical approaches for better understanding evolutionary mechanisms of genitalia.

## Materials and Methods

### Autofluorescence composition of the male flagellum and female spermathecal duct

The autofluorescence composition of the flagellum was investigated with the confocal laser scanning microscope Zeiss LSM 700 (Zeiss, Germany). For this purpose, beetles *Cassida rubiginosa* (Chrysomelidae) were collected in Jena (Germany) in May 2014 and in June 2015, kept in plastic jars with fresh leaves of thistles *Cirsium* spp., (Asteraceae) and grew to the next generation of adults. The shape and measurements of the male flagellum and female spermathecal duct of this species had reported in Filippov *et al*.^24^. Four males and three females were analysed. Of the treated samples, one male and female were analysed simultaneously to compare the intensity of autofluorescences between them. The flagella and spermathecal ducts were freshly dissected in the 1× phosphate butter solution (pH 7.4) (Carl Roth, Germany), and embedded in glycerine overnight. Subsequently, they were mounted on glass slides, and their autofluorescences were visualized, as described by Michels and Gorb^31^. The wave lengths of lasers 405, 488, 555, and 639 nm laser lines were used, and we applied a band-pass emission filter 420–480 and long pass emission filters transmitting light with wavelengths of ≥490, ≥560, and ≥640, respectively. Then we visualized the emitted wavelengths 420–480, ≥490, ≥560, and ≥640 with blue, green, red and red, respectively. All samples were observed using 10-fold objective lens with different digital zoom.

### Numerical model

Here we have developed further a numerical model previously proposed in Filippov *et al*.^24^. The model basically consists of two conceptual components: an elastic uncompressible fiber (flagellum), which can move inside an elastic channel. The transversal rigidity of the fiber and elasticity of the channel are adjustable. The flagellum motion is generated by pushing on its base by an external force. In the previous model, it was supposed that the female duct is generally stiffer than the flagellum, as it is suggested by our analysis of autofluorescence composition.

The flagellum was numerically constructed from a big number *N* (up to *N* = 10^5^) of elastically connected discrete segments. In the present study, we needed more accurate calculation than before and created more segments in comparison with the previous model^24^. Each segment has a length of *dr* ≤ 1 μm defined by a total length of the system *L* = 10 mm measured from the microscopic images for our model beetle, *Cassida rubiginosa*.

The flagellum model is provided with strong longitudinal stiffness 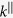 which prevents extension and compression of the practically rigid segments and two components of elastic forces 
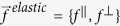
: 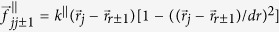, and 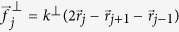, acting between the neighboring nodes 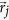. Longitudinal force, 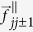, tends to conserve a distance between the nodes 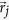 and 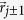 close to the equilibrium *dr*.

Like in the previous model^24^, we supposed that the transverse stiffness *k*^⊥^ = *const* is much weaker 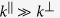 than the longitudinal one. In the real system, the flagellum seems to have different stiffness along its length (see below), which was taken into account in the current analysis. It means that *k*^⊥^ varies from segment to segment. From preliminary experiments we expect that *k*^⊥^ should be a nonlinear function, which is almost constant at the beginning and quickly falls down near the end. For definiteness we apply:


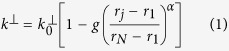


Here parameters 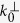 and *g* determine general rigidity and a rigidity gradient along the flagellum. In particular, at *g* = 0, large/small constant 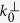 corresponds to the uniform hard/soft flagellum, respectively. If *g* = 0.99, then the tip of the flagellum is 100 times softer than its base. Using different combinations of the parameters, we analyzed four different variants of the system: constantly soft, constantly hard, almost constantly soft with the hard end, and almost constantly hard with the soft end. To reproduce the two last cases, we had used exponent α = 5.

Female duct along the coordinate 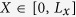 was defined by its spiral core:


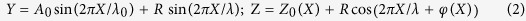


with the parameters R = *L*_*x*_/30; λ = *L*_*x*_/40, 0.1*L*_*x*_ < *A*_0_ < 0.5*L*_*x*_ and 0.2*L*_*x*_ < *λ*_0_ < 0.4*L*_*x*_. It has secondary structure with some number *N*_*knots*_ of knot points at positions *X*_*n*_, where direction of spiral rotation changes to a reverse one. The phase rotations were caused by the phase function φ(*X*) defined as a sum of step-like functions 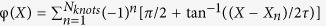 with factors (−1)^*n*^ = ±1 for mutually opposite phase rotations around even and odd nods, respectively.

The female spermathecal duct is not only helical but also convoluted^24^. However following the former paper^24^, we concentrated on the role of spiral and knot geometries and neglected all the variations of the spiral core *Y*_0_ = *A*_0_ cos (2*πX*/*λ*_0_) = 0 and limited ourselves by the straight spiraled-duct only. Such spirals having four knots *N*_*knots*_ = 4 and different combinations of the flagellum rigidities are shown in Figs 3 and 4. In each figure, the spiral axis of the female elastic channel is shown by a blue line. The position of the flagellum inside the female channel is shown by red curves and the flagellum tip is marked by a red circle. Numerically, the female duct was defined as an elastic confinement acting on every segment {*z*_*j*_(*x*_*j*_),*y*_*j*_(*x*_*j*_)} of the male flagellum by the returning forces: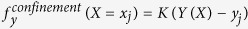 ; 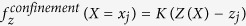, which are regulated by the elastic constant *K*.

For reflecting a probable uniform stiffness which is suggested by CLSM images, we fixed everywhere below, i.e. a general flagellum rigidity 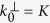 for the rigid and 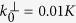 for the soft uniform flagellum (g = 0), respectively. Parameters *g* = 0.99 and 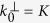 are chosen for the rigid gradient flagellum with the soft end and *g* = −99 and for 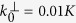 the soft flagellum with the hard end.

The presence of liquid inside the channel was taken into account by introducing a proportional to the velocity 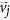 damping force: 
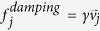
. This force was also added to the equations of motion of all the segments, which are the same as in previous study^24^:


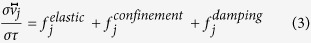


## Additional Information

**How to cite this article**: Filippov, A. E. *et al*. Stiffness gradient of the beetle penis facilitates propulsion in the spiraled female spermathecal duct. *Sci. Rep.* **6**, 27608; doi: 10.1038/srep27608 (2016).

## Supplementary Material

Supplementary Information

## Figures and Tables

**Figure 1:**
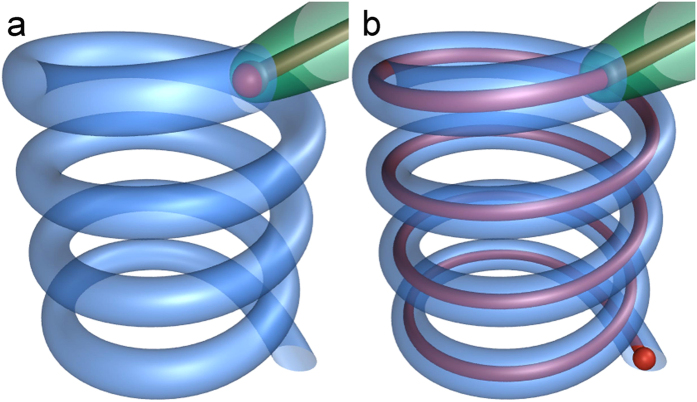
Schematics of female ducts (blue), male flagella (red) and male flagella guiding structures (green). Spheres on the male flagella show the tips of the male flagella. For simplicity, reversal turns (knots) are not depicted. (**a**) Mating is just stated; the tip of male flagella guiding structure is inserted to the female duct. (**b**) The male flagellum is fully inserted into the female duct.

## References

[b1] Eberhard W. G. Sexual selection and animal genitalia. (Harvard University Press, Cambridge, Massachusetts, USA, 1985).

[b2] Hosken D. J. & Stockley P. Sexual selection and genital evolution. Trends Ecol. Evol. 19, 87–93 (2004). 16701234 10.1016/j.tree.2003.11.012

[b3] Macagno A. L. M. . Shape - but not size - codivergence between male and female copulatory structures in *Onthophagus* beetles. PLoS One 6, e28893 (2011). 22194942 10.1371/journal.pone.0028893 PMC3237555

[b4] Rowe L. & Arnqvist G. Sexual selection and the evolution of genital shape and complexity in water striders. Evolution 66, 40–54 (2012). 22220863 10.1111/j.1558-5646.2011.01411.x

[b5] Simmons L. W. Sexual selection and genital evolution. Aust. Entomol. 53, 1–17 (2014).

[b6] Arnqvist G. & Rowe L. Sexual conflict and arms races between the sexes: a morphoogical adaptation for contral of mating in a female insect. Proc. R. Soc. Lond. B 261, 123–127 (1995).

[b7] Rodriguez V. Relation of flagellum length to reproductive success in male *Chelymorpha alternans* Boheman (Coleoptera: Chrysomelidae: Cassidinae). Coleopts. Bull. 49, 201–205 (1995).

[b8] Arnqvist G. & Danielsson I. Copulatory behavior, genital morphology and male fertilization success in water striders. Evolution 53, 147–156 (1999). 10.1111/j.1558-5646.1999.tb05340.x 28565197

[b9] House C. M. & Simmons L. W. Genital morphology and fertilization success in the dung beetle *Onthophagus taurus*: an example of sexually selected male genitalia. Proc. R. Soc. Lond. B 270, 447–455 (2003). 10.1098/rspb.2002.2266 PMC1691274 12641898

[b10] Cordoba-Aguilar A. Possible coevolution of male and female genital form and function in a calopterygid damselfly. J. Evol. Biol. 18, 132–137 (2005). 15669969 10.1111/j.1420-9101.2004.00796.x

[b11] Holwell G. I., Winnick C., Tregenza T. & Herberstein M. E. Genital shape correlates with sperm transfer success in the praying mantis *Ciulfina klassi* (Insecta: Mantodea). Behav. Ecol. Sociobiol. 64, 617–625 (2009).

[b12] Simmons L. W., House C. M., Hunt J. & Garcia-Gonzalez F. Evolutionary response to sexual selection in male genital morphology. Curr. Biol. 19, 1442–1446 (2009). 19664925 10.1016/j.cub.2009.06.056

[b13] van Lieshout E. & Elgar M. A. Longer exaggerated male genitalia confer defensive sperm-competitive benefits in an earwig. Evol. Ecol. 25, 351–362 (2010).

[b14] Sakurai G., Himuro C. & Kasuya E. Intra-specific variation in the morphology and the benefit of large genital sclerites of males in the adzuki bean beetle (*Callosobruchus chinensis*). J. Evol. Biol. 25, 1291–1297 (2012). 22536996 10.1111/j.1420-9101.2012.02517.x

[b15] Dougherty L. R., Rahman I. A., Burdfield-Steel E. R., Greenway E. V. & Shuker D. M. Experimental reduction of intromittent organ length reduces male reproductive success in a bug. Proc. R. Soc. Lond. B 282, 20150724 (2015). 10.1098/rspb.2015.0724 PMC4455817 25972470

[b16] Arnqvist G. Comparative evidence for the evolution of genitalia by sexual selection. Nature 393, 784–786 (1998).

[b17] Ah-King M., Barron A. B. & Herberstein M. E. Genital evolution: why are females still understudied? PLoS Biol. 12, e1001851 (2014). 24802812 10.1371/journal.pbio.1001851 PMC4011675

[b18] Villavaso E. J. Functions of the spermathecal muscle of the boll weevil, Anthonomus grandis. J. Insect Physiol. 21, 1275–1278 (1975).

[b19] Rodriguez V. Function of the spermathecal muscle in *Chelymorpha alternans* Boheman (Coleoptera: Chrysomelidae: Cassidinae). Physiol. Entomol. 19, 198–202 (1994).

[b20] Miller G. T. & Pitnck S. Sperm-female coevolution in *Drosophila*. Science 298, 1230–1233 (2002). 12424377 10.1126/science.1076968

[b21] Tanabe T. & Sota T. Both male and female novel traits promote the correlated evolution of genitalia between the sexes in an arthropod. Evolution 68, 441–452 (2013). 24116383 10.1111/evo.12288

[b22] Matushkina N. & Gorb S. N. Mechanical properties of the endophytic ovipositor in damselflies (Zygoptera, Odonata) and their oviposition substrates. Zoology 110, 167–175 (2007). 17400435 10.1016/j.zool.2006.11.003

[b23] Matsumura Y. . Two intromittent organs in *Zorotypus caudelli* (Insecta, Zoraptera): the paradoxical coexistence of an extremely long tube and a large spermatophore. Biol. J. Linn. Soc. 112, 40–54 (2014).

[b24] Filippov A. E., Kovalev A. E., Matsumura Y. & Gorb S. N. Male penile propulsion into spiraled spermathecal ducts of female chrysomelid beetles: A numerical simulation approach. J. Theor. Biol. 384, 140–146 (2015). 26341386 10.1016/j.jtbi.2015.08.002

[b25] Michels J., Gorb S. N. & Reinhardt K. Reduction of female copulatory damage by resilin represents evidence for tolerance in sexual conflict. J. R. Soc. Interface 12, 20141107 (2015). 25673297 10.1098/rsif.2014.1107 PMC4345479

[b26] Sentenská L., Pekár S., Lipke E., Michalik P. & Uhl G. Female control of mate plugging in a female-cannibalistic spider (*Micaria sociabilis*). BMC Evol. Biol. 15, 18 (2015). 25886749 10.1186/s12862-014-0278-9 PMC4327802

[b27] Willkommen J., Michels J. & Gorb S. N. Functional morphology of the male caudal appendages of the damselfly *Ischnura elegans* (Zygoptera: Coenagrionidae). Arthropod Struct. Dev. 44, 289–300 (2015). 25882740 10.1016/j.asd.2015.04.002

[b28] Rodriguez V., Windsor D. M. & Eberhard W. G. Tortoise beetle genitalia and demonstrations of a sexually selected advantage for flagellum length in *Chelymorpha alternans* (Chrysomelidae, Cassidini, Stolaini). In: New Developments in the Biology of Chrysomelidae (eds Jolivet P., Santiago-Blay J. A. & Schmitt M.), 739–748 (AcademicPublisher, the Hague, 2004).

[b29] Peisker H., Michels J. & Gorb S. N. Evidence for a material gradient in the adhesive tarsal setae of the ladybird beetle *Coccinella septempunctata*. Nat. Commun. 4, 1661 (2013). 23552076 10.1038/ncomms2576

[b30] Gorb S. N. & Filippov A. E. Fibrillar adhesion with no clusterisation: functional significance of material gradient along adhesive setae of insects. Beilstein J. Nanotechnol. 5, 837–845 (2014). 24991520 10.3762/bjnano.5.95 PMC4077360

[b31] Michels J. & Gorb S. N. Detailed three-dimensional visualization of resilin in the exoskeleton of arthropods using confocal laser scanning microscopy. J. Microsc. 245, 1–16 (2012). 22142031 10.1111/j.1365-2818.2011.03523.x

[b32] Gorb S. N. & Beutel R. G. Evolution of locomotory attachment pads of hexapods. Naturwissenschaften 88, 530–534 (2001). 11824227 10.1007/s00114-001-0274-y

[b33] Gorb S. N. . Structural design and biomechanics of friction-based releasable attachment devices in insects. Integr. Comp. Biol. 42, 1127–1139 (2002). 21680397 10.1093/icb/42.6.1127

[b34] Tadler A. Selection of a conspicuous male genitalic trait in the seedbug *Lygaeus simulans*. Proc. R. Soc. Lond. B 266, 1773–1777 (1999).

[b35] Kamimura Y. Promiscuity and elongated sperm storage organs work cooperatively as a cryptic female choice mechanism in an earwig. Anim. Behav. 85, 377–383 (2013).

[b36] Brennan L. R. . Coevolution of male and female genital morphology in waterfowl. PLoS One 5, e418 (2007). 17476339 10.1371/journal.pone.0000418 PMC1855079

