# Supplementary material for: Stiffness gradient of the beetle penis facilitates propulsion in the spiraled female spermathecal duct
Source: Sci Rep. 2016 Jun 23;6:27608. doi: 10.1038/srep27608 (PMC4918010; doi:10.1038/srep27608)
Supplement: Supplementary Information [file srep27608-s1.pdf]

Stiffness gradient of the beetle penis facilitates propulsion in the spiraled female spermathecal duct” by A.E. Filippov, Y. Matsumura, A.E. Kovalev, and S.N. Gorb

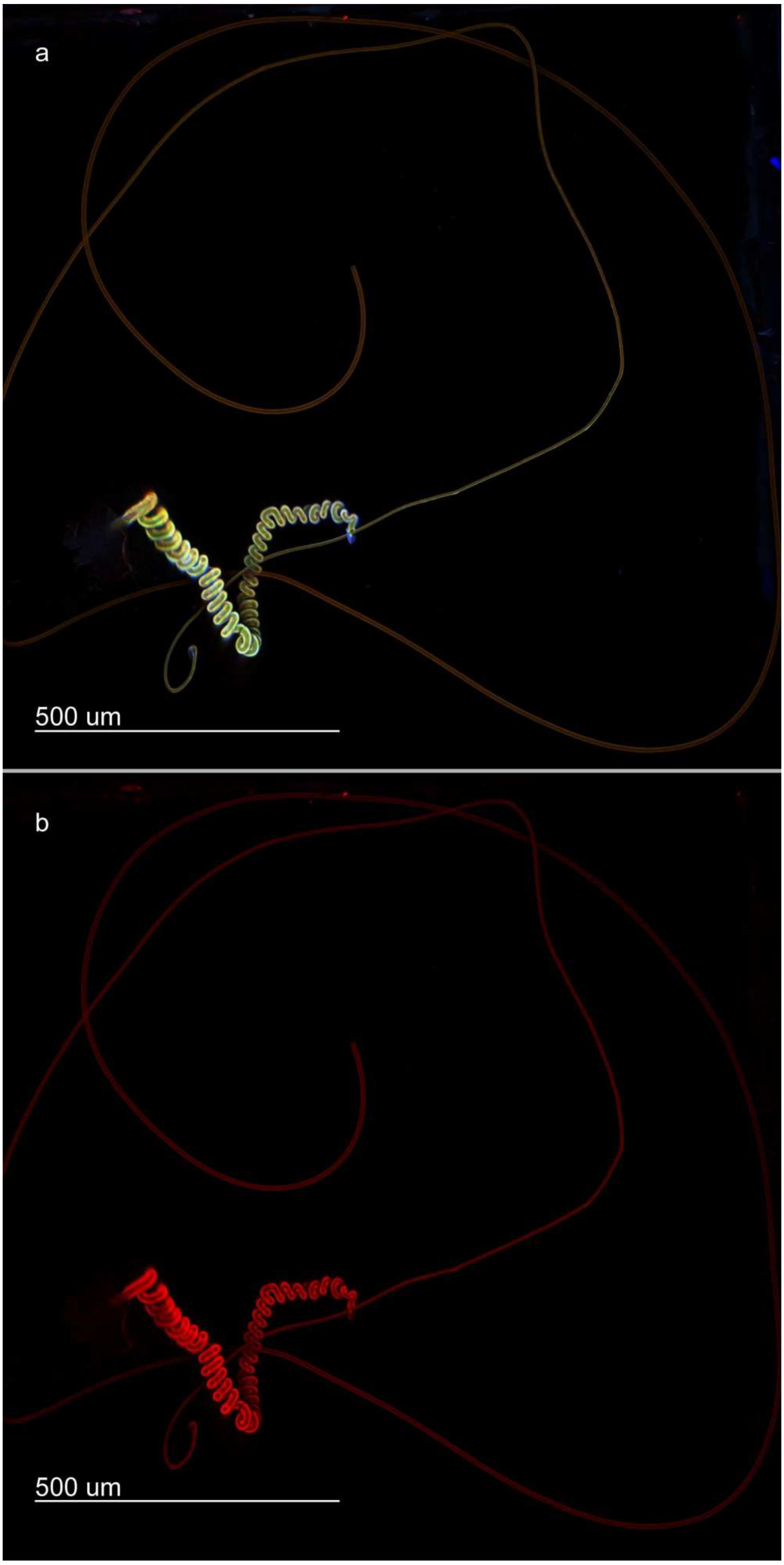
a
500 um
b
500 um

Supplementary Fig.1. Confocal laser scanning micrographs of the flagella and spermathecal duct of *Cassida rubiginosa*. a: All four autofluorescences which we used are shown. b: Only red autofluorescences are shown.
